# O01 - Validation of asthma and eczema in population-based Swedish health registers

**DOI:** 10.1186/2045-7022-4-S1-O1

**Published:** 2014-03-14

**Authors:** Anne K Örtqvist, Cecilia Lundholm, Björn Wettermark, Jonas F Ludvigsson, Weimin Ye, Catarina Almqvist

**Affiliations:** 1 Karolinska Institutet, Stockholm, Sweden

## Background

Valid measures of asthma and eczema in epidemiological studies may be a challenge. Population-based registers can be useful as they do not rely on recall and allow for follow-up. Our aim was to ascertain if asthma/eczema medication is a suitable proxy for asthma/eczema and to validate register-based asthma diagnoses.

## Method

Data were retrieved on all 0-17-year-old individuals with reported asthma/eczema medication in the Swedish Prescribed Drug Register (SPDR) and/or an asthma diagnosis in the National Patient Register (NPR) between 2005-2009 (N=121,944). Medical records retrieved from prescribing units, for 2,250 randomly selected individuals, were reviewed to estimate the proportion of individuals with 1) asthma/eczema medication in the SPDR who had a doctor diagnosis of asthma/eczema and/or; 2) fulfilled predefined criteria of asthma by the Swedish Paediatric Society (positive predictive value, PPV); 3) an asthma diagnosis in the NPR verified as asthmatics by predefined criteria.

## Results

PPV for asthma medication as a proxy for a doctor diagnosis of asthma was 0.68 (95%CI: 0.64-0.72) in pre-school children (0-4.5 years) and 0.89 (95%CI: 0.85-0.92) in school-age children (>4.5-17 years). The corresponding PPV for predefined criteria of asthma was 0.75 (95%CI: 0.70-0.78) and 0.94 (95%CI: 0.91- 0.96) in pre-school children and school-age children respectively. Almost all (99%) of school-age children and 78% of pre-school children with an asthma diagnosis in the NPR were verified as asthmatics. PPV for eczema medication as a proxy for an eczema diagnosis was estimated to 0.45 (95% CI: 0.38-0.51) in children 0-17 years.

## Conclusion

Asthma medication is the SPDR is a suitable proxy for asthma in older children; the same approach is insufficient for eczema. Furthermore, the quality of asthma diagnoses in the NPR is high. This validation study of two Swedish registers opens for future large nation-wide studies on asthma.

